# Comparative genomic and transcriptional analyses of CRISPR systems across the genus *Pyrobaculum*

**DOI:** 10.3389/fmicb.2012.00251

**Published:** 2012-07-13

**Authors:** David L. Bernick, Courtney L. Cox, Patrick P. Dennis, Todd M. Lowe

**Affiliations:** ^1^Department of Biomolecular Engineering, University of California, Santa CruzCA, USA; ^2^Janelia Farm Research Campus, Howard Hughes Medical Institute, AshburnVA, USA

**Keywords:** *Pyrobaculum*, CRISPR, sRNA, crRNA, repeat, RNAseq

## Abstract

Within the domain Archaea, the CRISPR immune system appears to be nearly ubiquitous based on computational genome analyses. Initial studies in bacteria demonstrated that the CRISPR system targets invading plasmid and viral DNA. Recent experiments in the model archaeon *Pyrococcus furiosus* have uncovered a novel RNA-targeting variant of the CRISPR system. Because our understanding of CRISPR system evolution in other archaea is limited, we have taken a comparative genomic and transcriptomic view of the CRISPR arrays across six diverse species within the crenarchaeal genus *Pyrobaculum*. We present transcriptional data from each of four species in the genus (*P. aerophilum*, *P. islandicum*, *P. calidifontis*, *P. arsenaticum*), analyzing mature CRISPR-associated small RNA abundance from over 20 arrays. Within the genus, there is remarkable conservation of CRISPR array structure, as well as unique features that are have not been studied in other archaeal systems. These unique features include: a nearly invariant CRISPR promoter, conservation of direct repeat families, the 5′ polarity of CRISPR-associated small RNA abundance, and a novel CRISPR-specific association with homologues of *nurA* and *herA*. These analyses provide a genus-level evolutionary perspective on archaeal CRISPR systems, broadening our understanding beyond existing non-comparative model systems.

## Introduction

CRISPR immunity systems, like the vertebrate adaptive immune system (Boehm, 2011), include mechanisms to adapt to new pathogens, surveillance methods for detecting previously encountered pathogens, and means to inactivate those pathogens. In the case of the CRISPR system, the targeted molecule is a nucleic acid sequence, and the sequence of events moves from adaptation, where the invading nucleic acid sequence is recognized and acquired, to expression, where the CRISPR-specific small RNA recognition molecules (crRNA) are transcribed, processed and loaded by the CAScade complex of CRISPR-specific proteins (Brouns et al., 2008; Jore et al., 2011). The third phase, interference, is initiated upon detection of a targeted nucleic acid sequence and results in specific inactivation of the recognized nucleic acid from the invading “pathogen.” DNA of viral or plasmid origin has been shown to be the target of CRISPR defense in bacteria (Barrangou et al., 2007; Marraffini and Sontheimer, 2008) and the archaeon *Sulfolobus solfataricus* (Manica et al., 2011). RNA sequences are targeted in the CRISPR system present in *Pyrococcus furiosus* (Hale et al., 2009, 2012), opening the possibility of endogenous targeting of messenger RNA sequence.

Most archaeal and many bacterial genomes contain one or more loci that encode the CRISPR system. Each CRISPR locus consists of an array of short DNA sequences, and frequently includes a cluster of CRISPR-associated (CAS) protein coding genes (Haft et al., 2005). The DNA arrays are composed of a leader sequence, followed by a set of 24–47 nucleotide (nt) direct repeats (DR) that form the delimiting punctuation of the array. The sequences between DR, termed spacers, are found to be 26–72 nt in length and encode small RNAs that are the stored immune memory for the system. The transcriptional promoter for the array is likely to be encoded within the leader sequence (Haft et al., 2005; Lillestol et al., 2009; Horvath and Barrangou, 2010). In *Escherichia coli*, the specific promoters for the array and associated CAS genes have been identified (Pul et al., 2010).

CRISPR arrays are dynamic structures, some containing only a single sequence while others may be quite large; for example, crispr4 in *Metallosphaera sedula* is over 10,000 nt in length and contains over 160 spacer sequences (Grissa et al., 2007). The genomes of most strains of *Methanococcus maripaludis* contain only one CRISPR array locus whereas the genome of strain S2 has no CRISPR array present. In contrast, the genomes of Methanocaldococcus strains encode between seven and 20 individual CRISPR arrays. In *Sulfolobus*, recent work has shown that selective pressure can be introduced *in vivo*, which results in deletion of genomic loci containing all or part of the CRISPR/CAS system (Gudbergsdottir et al., 2011).

Individual spacer elements in CRISPR arrays are acquired in the adaptation phase, during exposure to an invading genetic element. Evidence from surviving, phage-challenged cells shows an addition of one or more spacer sequences at the leader-proximal end of the array. These new spacer sequences are identical to phage sequence, can be from either phage genome strand, and confer immunity to survivor progeny (Barrangou et al., 2007). During this spacer acquisition phase, the target sequence is integrated into the array, likely through the action of CAS1, CAS2, and possibly other CAS proteins (for example, CSN2 in the *Streptococcus thermophilus* Type II system). This adaptation process only requires a single direct repeat in the array (Yosef et al., 2012). It is unclear if the acquired DNA spacer is derived directly from invading DNA, or if the DNA spacer is a copy produced during the adaptation process.

The mechanism of immunity is still incompletely understood, but immunity is dependent on CAS genes (Barrangou et al., 2007; Brouns et al., 2008), usually located near one or more CRISPR arrays. Early studies showed that four CAS genes (*cas1–4*) were frequently associated with CRISPR arrays (Jansen et al., 2002; Haft et al., 2005). A role in CRISPR adaptation (acquisition of new spacers) has been proposed for *cas1* and *cas2* (Wiedenheft et al., 2012). Potentially, CAS4 is also involved during the acquisition phase; this hypothesis is based on the frequent *cas4* genomic proximity to *cas1* (Makarova et al., 2011).

The CAS genes have recently been reclassified into three main families based on gene content and mode of action of the associated system (Makarova et al., 2011). In Type I, II, and III-A CRISPR systems (Makarova et al., 2011), the target of the CRISPR immunity system is invading DNA (Marraffini and Sontheimer, 2008). In contrast, Type III-B systems target RNA instead of DNA (Hale et al., 2009, 2012). Type I systems have been studied in both bacteria and archaea, and have recently yielded low-resolution structures of the multimeric CAScade complex in both *E. coli* (Jore et al., 2011; Wiedenheft et al., 2011) and in the archaeon *Sulfolobus solfataricus* (Lintner et al., 2011). In Type I systems, the CAScade complex is required for maturing of CRISPR RNA (crRNA) that guide protective immunity during subsequent invasion by foreign DNA elements. This crRNA-enabled complex is also responsible for surveillance and eventual interference by recruiting additional CAS proteins (Wiedenheft et al., 2011). The primary transcript of the CRISPR array, pre-crRNA, is cleaved within the DR to generate the individual crRNA segments. In the Sulfolobus variant of CAScade, CAS6 is responsible for cleavage of pre-crRNA, while in *E. coli* this role is carried out by CAS6e, also known as CasE (Brouns et al., 2008). The short RNA segments that are released from pre-crRNA processing retain an 8 nt 5′ “handle” sequence from the upstream DR as part of the mature crRNA (Brouns et al., 2008). Processing of pre-crRNA transcripts in *Sulfolobus* has been reported to proceed from the 3′ distal end toward the 5′ leader sequence (Lillestol et al., 2009). It is unclear how this 3–5′ directionality is established, given the site-specific endonucleolytic nature of CAS6 (Carte et al., 2008).

The Type III-B RNA-targeting CRISPR systems have been investigated in *Pyrococcus furiosus* (Hale et al., 2009, 2012) and in *Sulfolobus solfataricus* (Zhang et al., 2012). These systems include the cmr family of CAS genes along with the nearly ubiquitous *cas1*, *cas2*, and *cas6*. The *cmr* complex is composed of the protein products of *cmr1*, *cas10*, and *cmr3–cmr6*, plus the *cas6*-derived crRNA. In *Sulfolobus*, an additional *cmr* component, *cmr7*, joins the complex.

All CRISPR systems examined to date load crRNAs with 5′ OH ends, although the crRNA length and mature state of the 3′ end varies by CRISPR type and by species. We have therefore utilized a cloning strategy that is independent of 5′ end chemistry and partially independent of 3′ end chemistry.

In this study, we show linkage of CAS protein types with families of CRISPR arrays, conservation of CRISPR array elements across the genus, a novel *nurA-csm6-herA* gene cluster associated with *Pyrobaculum* CRISPR arrays, and provide transcriptional support for polarity in crRNA abundance.

## Methods

### Culture conditions

*P. aerophilum* cells were grown anaerobically in media containing 0.5 g/L yeast extract, 1X DSM390 salts, 10 g/L NaCl, 1X DSM 141 trace elements, 0.5 mg/L Fe(SO_4_)_2_(NH_4_)_2_, pH 6.5, with 10 mM NaNO_3_. *P. islandicum* and *P. arsenaticum* cells were grown anaerobically in media containing 10 g/L tryptone, 2 g/L yeast extract, 1X DSM390 salts, 1X DSM88 trace elements, and 20 mM Na_2_S_2_O_3_. *P. calidifontis* cells were grown aerobically in 1L flasks using 500 ml media containing 10 g/L tryptone, 2 g/L yeast extract, 1X DSM88 trace metals, 15 mM Na_2_S_2_O_3_, pH 6.8, loosely capped with moderate shaking at 125 rpm. Anaerobic cultures were grown in 2L flasks with 1L media, prepared under nitrogen with resazurin as a redox indicator at 0.5 mg/L; 0.25 mM Na_2_S was added as a reductant. All cultures were grown at 95C to late log or stationary phase, monitored at OD_600_.

The 10X DSM390 salts are comprised of (per liter ddH_2_O) 1.3 g (NH_4_)_2_SO_4_, 2.8 g KH_2_PO_4_, 2.5 g MgSO_4_·7H2O. The 100X DSM88 trace metal solution is comprised (per liter 0.12N HCl), 0.9 mM MnCl_2_, 4.7 mM Na_2_B4O_7_, 76 μM ZnSO_4_, 25 μM CuCl_2_, 12.4 μM NaMoO_4_, 18 μM VOSO_4_, 6 μM CoSO_4_. The 100X DSM141 trace metal solution is comprised of 7.85 mM Nitrolotriacetic acid, 12.2 mM MgSO_4_, 2.96 mM MnSO_4_, 17.1 mM NaCl, 0.36 mM FeSO_4_, 0.63 mM CoSO_4_, 0.68 mM CaCl_2_, 0.63 mM ZnSO_4_, 40 μM CuSO_4_, 42 μM KAl(SO_4_)_2_, 0.16 mM H_3_BO_3_, 41 μM Na_2_MoO_4_, 0.1 mM NiCl_2_, 1.14 μM Na_2_SeO_3_.

### cDNA library preparation

The cDNA libraries were prepared using small RNA fractions collected from cells grown to stationary and exponential phase, using methods previously described (Bernick et al., 2012), with brief details given in Results. These two preparations were constructed for each of *P. aerophilum, P. islandicum, P. arsenaticum*, and *P. calidifontis* cultures, yielding a total of eight cDNA libraries.

The 3′ end chemistries of crRNA have been reported as either 2–3′ cyclic phosphate (Hale et al., 2012; Jore et al., 2011), or as 3′ OH (Hatoum-Aslan et al., 2011; Zhang et al., 2012). Under the acidic conditions (pH 5) used in RNA preparation in this study, we expect an equilibrium population of 3′ OH terminated RNA to exist under either scenario, providing a cloning method that is semi-independent of 3′ end chemistry.

### Sequencing and read mapping

Sequencing was performed using a Roche/454 GS FLX sequencer, and the GS emPCR Kit II (Roche). Sequencing reads in support of this work are provided online via the UCSC Archaeal Genome Browser (http://archaea.ucsc.edu) (Chan et al., 2012).

Reads that included barcodes and sequencing linkers were selected from the raw sequencing data and used to identify reads from each of the eight pooled cDNA libraries. Reads were further consolidated, combining identical sequences with associated counts for viewing with the Archaeal Genome Browser. Reads were mapped to the appropriate genome [*P. aerophilum* (NC_003364.1); *P. arsenaticum* (NC_009376.1); *P. calidifontis* (NC_009073.1); *P. islandicum* (NC_008701.1); *P. oguniense* (NC_016885.1); *P. neutrophilum* (*T. neutrophilus*: NC_010525.1)] using BLAT (Kent, 2002), requiring a minimum of 90% identity (-minIdentity), a maximal gap of 3 (-maxIntron) and a minimum score (matches minus mismatches) of 16 (-minScore) using alignment parameters for this size range (-tileSize = 8-stepSize = 4). Reads that mapped equally well to multiple positions in the genome were excluded from this study. The remaining, uniquely mapped reads were formatted and visualized as BED tracks within the UCSC Archaeal Genome Browser.

### Computational prediction of orthologous gene clusters

Computational prediction of orthologous groups was established by computing reciprocal best BLASTP (Altschul et al., 1990) (RBB) protein coding gene-pairs among pairs of four *Pyrobaculum* species. When at least three RBB gene-pairs select the same inter-species gene set (for example A pairs with B, B pairs with C, and C pairs with A), the cluster is considered an orthologous gene cluster.

### CRISPR array mapping

Arrays were predicted using CRISPRfinder (Grissa et al., 2007). Arrays were merged in some cases-based on sequencing data evidence.

## Results

### CRISPR/CAS protein families

**Figure 1:**
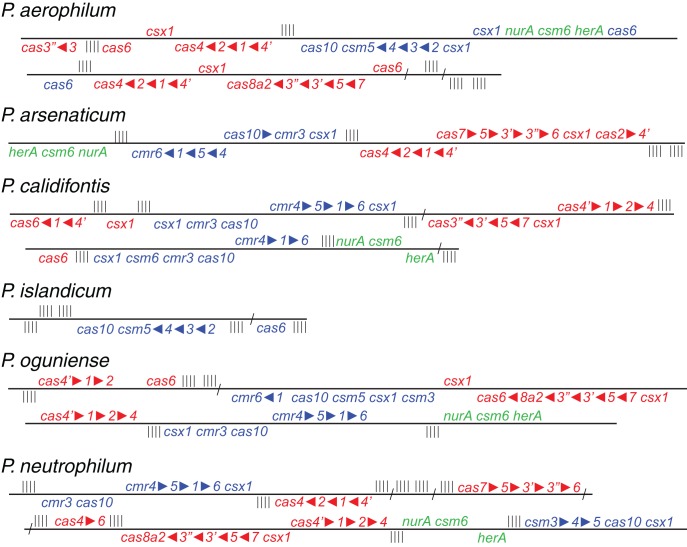
**Genomic arrangement of CRISPR modules within *Pyrobaculum* species.** Colors indicate Type I CAS modules (red), Type III CAS modules (blue) and CRISPR-associated *nurA-csm6-herA* clusters (green). CRISPR DNA arrays represented as vertical bars. Arrangements of multiple genes in the same CAS family are indicated using filled triangles (*cas1-cas2* is indicated as *cas1*
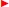
2). Genomic distances greater than 10 Kb are indicated using diagonal slashes (“/”). Gene strand is indicated relative to the solid black line for positive (above) and negative (below) orientations. Most *Pyrobaculum* species encode both Type I and III CAS modules; *P. islandicum* encodes only a Type III CAS module.

Three distinct types of CAS gene clusters exist within the six *Pyrobaculum* species examined (Figure 1 and Table A1) (Makarova et al., 2011). In most *Pyrobaculum* species, the Type I system is present, organized in submodules. Typically we find a submodule that includes: *cas1*, *cas2*, *cas4*, and a *cas4* variant herein referred to as *cas*4′, previously described as *csa1* (Haft et al., 2005) (submodule abbreviation *cas4*′-*1*-*2*-*4*). A second submodule is found nearby, comprising *cas6*, *cas7*, *cas5*, *cas3*′, *cas3*″, and *cas8a2* (abbreviated *cas6-7-5-3*′−*3*″-*8a2*) (Figure 1). With the exception of *P. islandicum*, each species in the genus has these submodules or close variants, and one or more submodules may be duplicated. In some cases, terminal members of the submodule may be relocated, such as *cas6* in *P. calidifontis* or *P. neutrophilum*. Type I subtypes are defined by the presence of specific genes: *cas8a1* or *cas8a2* (subtype I-A); *cas8b* (subtype I-B); *cas8c* (subtype I-C); *cas10d* (subtype I-D); *cse1* (subtype I-E); and *csy1* (subtype I-F) (Makarova et al., 2011). *P. aerophilum*, *P. oguniense*, and *P. neutrophilum* contain *cas8a2*, so fall within the definitive Type I-A subtype. *P. arsenaticum* and *P. calidifontis* do not appear to contain any recognized signature genes, so the subtype remains indeterminate. Notably, the Type I system is completely absent from *P. islandicum.*

A second CAS group, the Type III-B family of RNA-targeting CAS genes, is present in four *Pyrobaculum* species but not in *P. aerophilum* or *P. islandicum*. Again, this second family is present as submodules, with *cmr4*, *cmr5*, *cmr1*, and *cmr6* (*cmr4-5-1-6*) adjacent but on the opposite strand of the *cmr3-cas10* submodule. One or both of these submodules include *csx1*, and are currently classified as members of Type III-U (unclassified Type III). We find that *csx1* also appears in the Type I modules, so this suggests a broader role for *csx1* among *Pyrobaculum* CAS modules.

The third kind of module found in the genus, Type III-A (*csm*), appears to be complete in *P. aerophilum*, and is the only apparent CAS family found in *P. islandicum*. Previously, Makarova suggested that CRISPR adaptation for Type III families may require use of *cas1* and *cas2* in *trans* from a resident Type I family member (Makarova et al., 2011). However, this option is unavailable in *P. islandicum*, suggesting that adaptation for *Pyrobaculum* Type III systems may not require *cas1-cas2*. Possibly, an undescribed enzyme fulfills this role, or *P. islandicum* may have lost the ability to further adapt its CRISPR arrays.

Curiously, *csm6* is absent from *P. islandicum*, but is present in every other species examined in this study. This is notable because *csm6* would be expected to be part of the Type III-A system in *P. islandicum*, and would not be expected in species that do not encode a complete Type III-A module. Both *P. oguniense* and *P. neutrophilum* encode a portion of the Type III-A module (*csm3-csm5-cas10-csx1*) but both species are missing *csm2* and *csm4*. Where *csm6* is present, it is located next to a conserved paralog of *nurA* and *herA*; these genes are near a CRISPR array in species of *Pyrobaculum*, *Thermoproteus*, and *Vulcanisaeta*, suggesting that this arrangement is widespread among the Thermoproteales.

**Figure 2:**
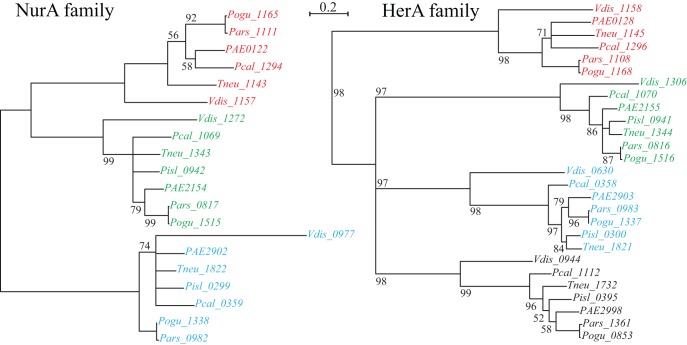
**NurA and HerA gene families in *Pyrobaculum* species.** *Pyrobaculum* species encode at least three *nurA-herA* gene-pairs, shown above to display parallel phylogenetic structure (left and right panels), with genomically adjacent genes matched by color. Within each gene clade (red, green or blue), the relationship of genes follows the expected species tree, indicating robust ortholog groups. The CRISPR-associated *nurA-herA* pairs (shown in red above) maintain the paired relationship throughout the Thermoproteales. An unmatched paralogous group of *herA* genes appears in black. The *Vulcanisaeta distributa nurA-herA* genes are provided as outgroups of the *Pyrobaculum* genus; note that only the CRISPR-associated *nurA-herA* pair (Vdis_1157-Vdis_1158) is collocated in *V. distributa*, further supporting the tight functional association. Bootstrap percentages are included in black (100% unless otherwise noted). Gene name prefixes: *Vdis—Vulcanisaeta distributa*; *PAE*—*Pyrobaculum aerophilum*; *Pars*—*P. arsenaticum*; *Pisl*—*P. islandicum*; *Pcal*—*P. calidifontis*; *Pogu*—*P. oguniense*; *Tneu*—*P. neutrophilum*. Multiple alignment of amino-acid sequences was performed using Clustal-Omega (v 1.04), maximum-likelihood trees were computed using Tree-puzzle (v 5.02), and the final tree was visualized using newicktops (v 1.0), available through the Pasteur Institute's Mobyle portal (http://mobyle.pasteur.fr/).

The *nurA-herA* protein complex is comprised of a 5–3′ DNA exonuclease (*nurA*) and a bidirectional helicase (*herA*) with probable involvement in homologous recombination (HR) (Constantinesco et al., 2004). HR processing requires a 3′ single stranded DNA (ssDNA) resection of chromosomal ends resulting from a double-strand break, and in thermophilic archaea, that resection is carried out by the helicase-nuclease complex of HerA-NurA (Blackwood et al., 2012). In most *Pyrobaculum* spp., there are three or more paralogs of this gene-pair, one of which is clustered with *csm6* and near a CRISPR array (Figure 1). Computationally predicted orthologs of the CRISPR-associated *nurA*-*herA* genes (RBB) show that this pair has been retained throughout the *Pyrobaculum* genus and more broadly among the Thermoproteales (Figure 2 and Table A2). In *P. islandicum*, however, the CRISPR-associated *nurA-herA* pair and *csm6* are absent. We propose that the *nurA-csm6-herA* complex may be associated with adaptation in *Pyrobaculum* species. Three possibilities arise from this proposal: (1) adaptation in *P. islandicum* may have been lost; (2) adaptation in *P. islandicum* may occur using an alternative mechanism, possibly one of the *nurA-herA* paralogs; or (3) the *nurA-csm6-herA* trio may only be required in Type I CRISPR systems (Yosef et al., 2012).

### CRISPR arrays

We have characterized three distinct families of CRISPR arrays present among six sequenced *Pyrobaculum* genomes (Table 1). These three families are defined by the sequences central to the DR and typically contain an A-rich core of 3–5 nt. These central motifs are flanked by short reverse complement (RC)-palindromes. The DR is terminated by an 8 nt-long sequence that becomes the 5′ handle of the mature crRNAs (Brouns et al., 2008). The various *Pyrobaculum* species encode between four and seven CRISPR arrays within their respective genomes. Except for *P. islandicum*, all species contain one or more representatives of family I and at least one additional representative from family III.

**Table 1:** ***Pyrobaculum* direct repeat (DR) families**.

**Type**	**5′ motif**	**p**	**core**	**p′**		**5′ crRNA handle**	***P. aerophilum***	***P. arsenaticum***	***P. islandicum***	***P. neutrophilum***	***P. calidifontis***	***P. oguniense***
I	GAAT	CTC	AAAAA	GAG	G	ATTGAAAG	1	3				2
	GAAT	CTC	AAGAA	GAG	G	ATTGAAAG					4	
	GAAT	CTC	AAAGA	GAG	G	ATTGAAAG				2		
	GAAT	CTC	AAGTT	GAG	G	ATTGAAAG				2^*^		
	GATT	CTC	AGATA	GAG	A	TTTGAAGG				1		
III-B	GAGAAT	CCCC	AAA	GGGG		GTAGAAAC					3	
III-A	CCAGAA	ATC	AAAA	GAT	A	GTTGAAAC	4					1
III	CCAGAA	ATC	AAAA	GAT	A	GTAGAAAC			5	5		
III-B	GTCAAA	ATC	AAAA	GAT	A	GTTGAAAC		1				1^*^

Alignment of direct repeats across known Pyrobaculum species. Pyrobaculum DR sequences include a variable length 5′ motif, two short inverted repeats (p and *p*′) surrounding an A-rich core region, followed by one or zero nucleotides, and ending in what will become the 5′ handle of processed crRNA. Identical motifs are shown in gray below first instance. Numbers in species columns refer to number of CRISPR arrays harboring DRs of that type. Asterisk (*) indicates DR mixing has occurred in one of the CRISPR arrays in this species. The associated CAS type is inferred by adjacency to an array using that DR family.

A single array may include multiple families of DR sequences, as found in *crispr1* of *P. oguniense* and *crispr5* of *P. neutrophilum*. In these unusual cases, the DRs are clustered; for example in the *P. neutrophilum* case, the type I DR array begins with 11 repeats using the “AAGTT” core, followed by a set of four repeats mixing “AAAAA” with “AAAGA” cores, and terminating with three “AAAGA” core repeats. In *P. oguniense*, *crispr1* has eight repeats with a 5′ motif of “GTCAAA” and five repeats with a 5′ motif of “CCAGAA.” In both cases where DR mixing was observed, the array type (based on CAS proteins) is maintained (Table 1). Previous studies in *E. coli* have shown that new DRs are added to an array during adaptation, by copying the first DR in the array (leader-proximal) (Yosef et al., 2012). We note that non-mixed arrays exist in *P. neutrophilum* whose leader-proximal repeats include the “AAAGA” and “AAGTT” cores. Potentially, DR mixing may come about through HR (duplication) events, or possibly by copying a leader-proximal DR from another array during adaptation.

A 5′ promoter-like sequence (AAAAACTTAAAAA) is ultra-conserved with only three single nt polymorphisms among all 37 CRISPR arrays in the six *Pyrobaculum* species studied. The same promoter-like element is also associated with some tRNA genes in these genomes. The sequence variation in the corresponding promoter elements for other genes is commonly much more diverse. This finding suggests that the invariant CRISPR promoter sequence is maintained either through strong purifying selection or through frequent gene-conversion (Liao, 2000).

CRISPR/CAS protein families appear to be associated with arrays of a given sequence family. This association is upheld to the CAS type, but does not extend to the subtype. For example, in *P. islandicum*, the only CAS family present is Type III-A (Figure 1) and the five encoded arrays in that species use a single DR type (Table 1). This same DR is also found in *P. neutrophilum* next to a Type III-B CAS cluster. In a second example, the mixed *crispr1* in *P. oguniense* is made up of DRs associated with Type III-A CAS clusters as found in *P. aerophilum*, and Type III-B CAS clusters, as found in *P. arsenaticum*. Both of these examples demonstrate the association of CAS types (not subtypes) with CRISPR array families in the *Pyrobaculum* genus.

Pre-crRNA transcripts are subjected to endonucleolytic processing to yield individual crRNA sequences, which we detect within small-RNA libraries. Deep sequencing from four *Pyrobaculum* species yielded thousands of sequencing reads, representing between 3% (*P. arsenaticum*) and 20% (*P. islandicum*) of the total sequencing reads in the 20–70 nt size range (Table 2).

**Table 2:** **CRISPR crRNA abundance (counts) in *Pyrobaculum* species from each CRISPR array, measured during exponential (expo) and stationary (stat) growth phases**.

**Species**	**CRISPR id**	**Type**	**Size**	**expo**	**stat**	**total**
*P. aerophilum*	*crispr1*	III	13	361	146	507
	*crispr2*	III	17	342	91	433
	*crispr3*	I	80	1298	417	1715
	*crispr5*			degenerate array		
	*crispr7/6*	III	11	305	101	406
	sum			2306	755	3061
	Total RNA			17,785	13,042	30,827
	crispr%			13.0%	5.8%	9.9%
*P. arsenaticum*	*crispr2*	I	34	178	339	517
	*crispr3*	I	84	183	230	413
	*crispr4*			degenerate array		
	*crispr5*	III		degenerate array		
	*crispr6*	I	6	5	10	15
	sum			366	579	945
	Total RNA			14,854	16,352	31,206
	crispr%			2.5%	3.5%	3.0%
*P. islandicum*	*crispr1*	III	17	691	455	1146
	*crispr2*	III	14	635	349	984
	*crispr3*	III	2	627	586	1213
	*crispr4*	III	3	594	416	1010
	*crispr5*	III	34	2363	1661	4024
	sum			4910	3467	8377
	Total RNA			28,128	14,823	42,951
	crispr%			17.5%	23.4%	19.5%
*P. calidifontis*	*crispr1*	III	2	545	340	885
	*crispr2*	III	3	302	226	528
	*crispr3*	III	2	156	150	306
	*crispr4*	I	8	180	85	265
	*crispr5*	I	35	233	270	503
	*crispr6*	I	36	274	248	522
	*crispr7*	I	2	12	13	25
	sum			1702	1332	3034
	Total RNA			22,102	17,192	39,294
	crispr%			7.7%	7.7%	7.7%

**Figure 3:**
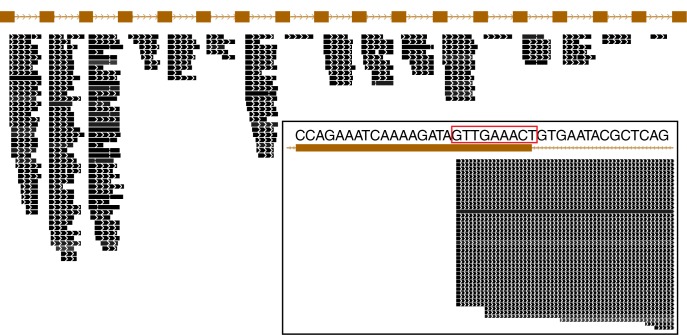
**Small RNA expressed from a typical CRISPR array in *Pyrobaculum aerophilum*.** The *crispr2* array is shown, depicting the near-identical repeat regions (brown rectangles) with intervening spacer sequences. Mature crRNA reads (black bars) that map uniquely to individual spacers within CRISPR arrays are shown with strand indicated by interior arrowheads. The 5′ ends of crRNA are sharply terminated and includes an 8-base sequence (5′ handle boxed in red) derived from the upstream DR (inset panel). Images generated from the UCSC Archaeal browser (Chan et al., 2012). CRISPR annotation derived from CRISPRFinder (Grissa et al., 2007).

**Figure 4:**
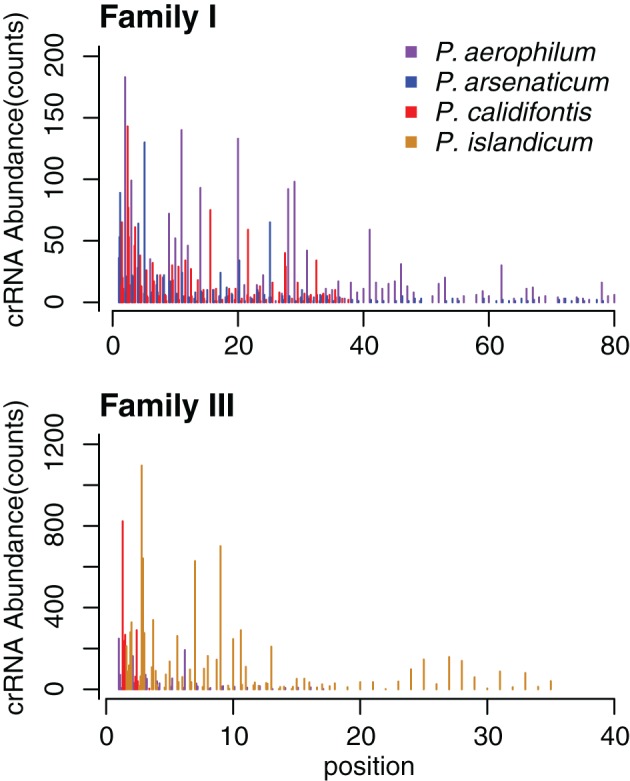
**CRISPR small RNA abundance density in *Pyrobaculum* CRISPR arrays by family.** Abundance is depicted on the vertical axes, in each species at every spacer position in the array. The horizontal axes plot crRNA genetic origin in terms of spacer number within CRISPR arrays, starting at the 5′ end of arrays. The abundance profile appears higher at the 5′ portion CRISPR arrays, with considerable variation deeper (3′) in the array. Multiple arrays of the same type within a species are plotted as adjacent bars by spacer position.

The abundance of individual crRNAs appears to be related to their position within the array (Figure 3). Abundance is generally highest when the spacer is located in the leader-proximal (5′) portion of the array, and decays distally (3′) (Figure 4), as seen in *Pyrococcus* (Hale et al., 2012). This pattern is evident in most *Pyrobaculum* arrays that contain more than five spacers. We also see significant variation in crRNA abundance against this decaying background pattern as described for *Sulfolobus* species (Zhang et al., 2012).

**Figure A1:**
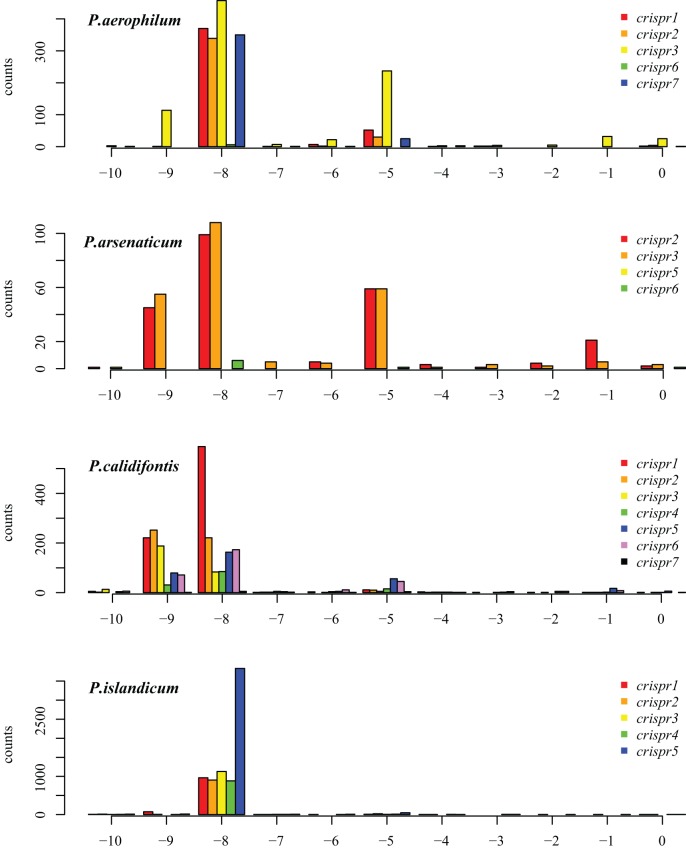
**Distribution of mapped 5′ ends of crRNA associated reads within CRISPR arrays in *P. aerophilum*, *P. arsenaticum*, *P. calidifontis*, and *P. islandicum*.** The majority of transcription sequencing reads begin at position −8 (relative to the beginning of the associated spacer (position 0). This finding implies that most crRNA associated sequencing reads include the 8 nucleotide 5′ handle sequence. A minority population of transcription reads begins at position −5. A third population of sequencing reads begin at position −9; these may be an artifact of the terminal transferase activity of MMLV derived reverse transcriptases. This activity most often yields a terminal cytosine residue to the 3′ end of the cDNA, yielding an implied “G” to the 5′ end of the sequencing read Chenchik et al. (1996).

The majority of terminal positions of sequencing reads found in *Pyrobaculum* species include an 8-base portion of the upstream DR at the 5′ end (Figure A1); this corresponds to the 5′ handle (Brouns et al., 2008) (Figure 3). We also see a minority population of sequencing reads that include a 5-base portion of the upstream DR (Figure A1), though these are not present in *P. islandicum*.

**Figure A2:**
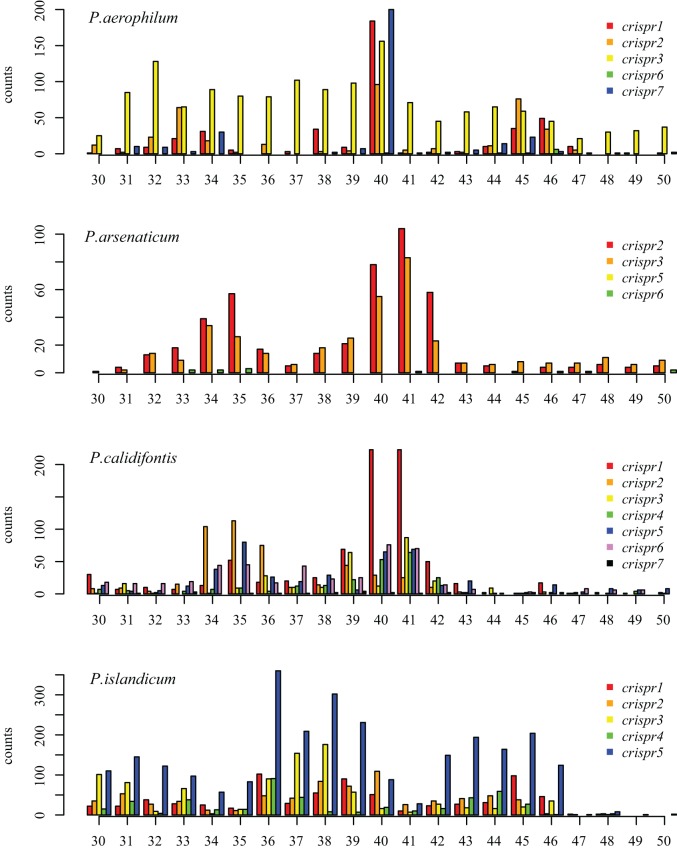
**Distribution of mapped 3′ ends of crRNA associated sequencing reads, relative to the 5′ end of the associated spacer.** This model proposes that cleavage of the 3′ end of crRNA associated reads utilizes a ruler-mechanism measured from the upstream DR. In *P. aerophilum*, *P. arsenaticum*, and *P. calidifontis*, the majority population of crRNA associated sequencing reads have a 3′ end centered around positions 40–41. A second minority population has a 3′ end centered around positions 32–35. In *P. islandicum*, the majority 3′ end is centered at positions 36–38, and a second minority 3′ end is centered around positions 42–46.

**Figure A3:**
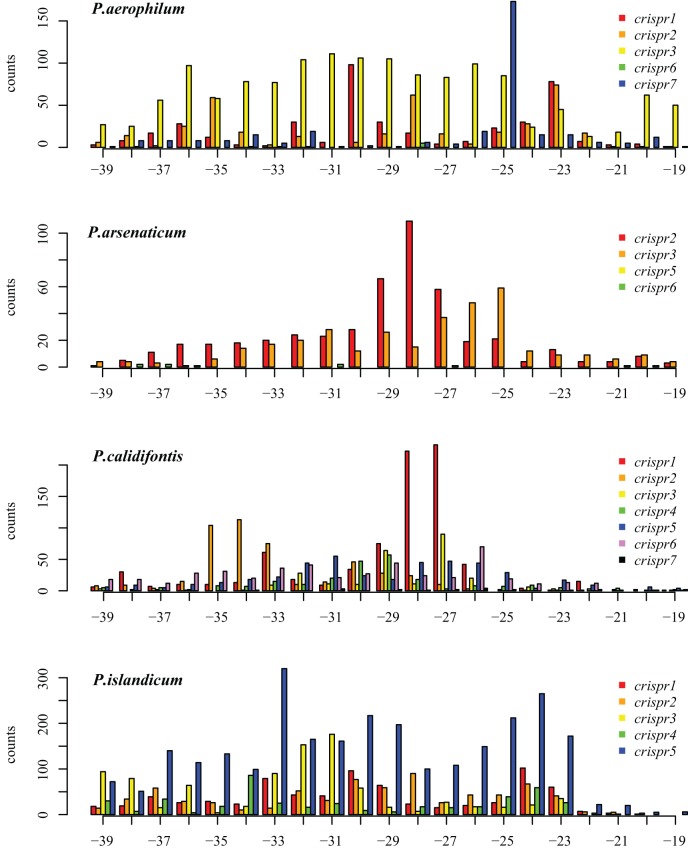
**Distribution of mapped 3′ ends of crRNA associated sequencing reads, relative to the downstream Direct repeat.** (position 0 is the start of the downstream spacer). This alternative model proposes that the downstream DR establishes the 3′ cut site. In *P. aerophilum*, the major population of 3′ ends shown for crispr 1–3 (Figure A2, position 40) is much more diffuse when measured in relation to the down stream direct repeat; this suggests that the 3′ cleavage of crRNA better modeled using the upstream DR as reference rather than the alternative, downstream DR reference. A single spacer region dominates abundance of crRNA in *P. aerophilum*.crispr7; this abundance provides the peak at −25 (corresponding to position 40 in Figure A2). In the remaining species, an attempt to distinguish between models of the underlying 3′ cleavage position was inconclusive.

We tested two models for 3′ maturation considering an upstream DR ruler-mechanism as seen in *Staphylococcus* species (Hatoum-Aslan et al., 2011), and a wrap-around model involving the downstream DR, as described for *Pyrococcus furiosus* (Wang et al., 2011). Because spacer sizes are not uniform in these species, we examined 3′ processing by testing distributions of 3′ end positions as measured from either the upstream DR or the downstream DR, under the assumption that spacer size variation would provide added noise to the incorrect model. Under the ruler-mechanism model, the 3′ distribution of end positions in *P. aerophilum*, *P. arsenaticum*, and *P. calidifontis* includes majority peaks at positions 40–41, and a minority peak at position 32 in *P. aerophilum* (Figure A2). Under the downstream DR based wrap-around model (Figure A3), *P. aerophilum* has a reduced peak at −25 (corresponding to position 40 in the ruler-mechanism model) and the minority peak is absent (seen previously at position 32). We consider this evidence as consistent with a ruler-mechanism for *P. aerophilum* CRISPR systems. In the remaining species, this analysis was inconclusive.

We find limited evidence for bidirectional CRISPR transcription as reported in *Sulfolobus* (Lillestol et al., 2009). Across all four of the *Pyrobaculum* species in the selected 16–70 nt size range, we see less than 1% of 15,417 CRISPR reads that map to the reverse strand of the array. Where those antisense reads are present, they appear to originate within the spacers and terminate at poly-T motifs within the DR regions. With the limited number of reverse reads seen in this size range, it appears that transcription from the opposite strand is either not processed down to the size range studied, or that reverse transcripts are much less abundant in *Pyrobaculum*. Potentially, this negative finding could be the result of the ubiquitous poly-A sequence present in every DR studied in this genus (Table 1). We anticipate that the poly-A sequence could mimic a poly-T terminator on the reverse strand, and thereby prevent significant reverse strand transcription.

## Discussion

Within CRISPR arrays, we see an overabundance of reads emanating from the 5′ proximal portion in larger arrays, where transcription from these arrays is likely initiated from a single promoter. The polarity is not perfect given that the abundance of some distal spacers is greater in comparison to more proximal spacer positions. Clearly, there are a number of mechanisms or phenomena that could contribute to crRNA abundance across the array, including: (1) simple stochastic termination of the pre-crRNA transcript, (2) differential efficiency in the endonucleolytic processing of individual crRNAs, (3) transcriptional polarity within the array, (4) differential stability of individual crRNAs, (5) selective recovery and amplification of certain crRNA sequences during library preparation, and (6) recently evolved changes in spacer content (gain or loss or rearrangements) between the reference genome strain and the cultured strains used in our RNA-seq experiments.

It is unknown which or how many of the six possibilities are most relevant, although our data do not equally favor all. If we consider a model of passive, stochastic termination of the primary transcript, we could explain the 5′ polarity but fail to account for the intermediate crRNA variation. Alternatively, a model where individual spacers are matured (excised) from pre-crRNA with varying efficiency might explain the variation in spacer abundance, but the 5′ polarity would be more difficult to accommodate. Instead, we tend toward a model that relies on coupling of pre-crRNA transcription with processing of the transcript, which might explain both polarity and the intermediate variation; for example, if transcription is aborted under conditions of limiting processing capability. We note that some bacterial systems make use of *rho*-mediated termination, coupling transcription and translation in a manner that aborts transcription under conditions of limiting polysomes; this process yields an abundance polarity favoring genes that are near the 5′ end of an operon transcript. Recently, operon polarity has been described in the archaeon *Thermococcus kodakaraensis* (Santangelo et al., 2008). In a polarity model that couples CRISPR pre-crRNA transcription with crRNA processing, we hypothesize that given a limitation in processing by the CRISPR CAScade complex (or *cmr*-processing complex), the pre-crRNA transcript might be prematurely aborted, yielding an abundance of 5′ crRNA. Compelling evidence exists for incremental, endonucleolytic processing of the primary transcript in other species (Brouns et al., 2008; Hale et al., 2008). Under this 5′ polarity model, we would expect to see both polarity as well as a degree of variation in individual spacer abundance, which seems to match our data the closest. This model is necessarily incompatible with 3–5′ directional processing that has been suggested previously (Lillestol et al., 2006).

Within the *Pyrobaculum* genus, one of the conserved *nurA-herA* clusters of syntenic orthologs is always found next to a CRISPR array (Figure 2). This cluster includes *csm6*, a gene classified with the Type III-A CRISPR/CAS family. In every case observed, *nurA-csm6* appear to be co-transcribed, in some cases with *herA*. The studied function of *nurA-herA* involves preparation of dsDNA ends as part of HR repair. If these genes participate in CRISPR processing, we suggest that they may be part of new spacer acquisition. That process requires the creation of a new DR and the integration of a novel spacer sequence into an existing array. Generally, this process yields an array with perfect copies, suggesting that the source of the novel DR sequence is an existing array element. In this model, a *nurA-herA* protein complex could provide the HR activity required to repair the array incision.

The phylogeny of the *nurA-herA* orthologous pairs suggests that they have been inherited vertically (Figure 2). Furthermore, a parsimonious interpretation of these gene trees indicates that the CRISPR-specific pair predates the divergence of *Pyrobaculum* species, and is well-represented across the Thermoproteaceae. The DR sequences that are in use throughout the *Pyrobaculum* are also remarkably conserved, with only three major sequence variants found, corresponding to the CAS proteins that make use of these structures. The structural conservation of the CAS operons is consistent across the *Pyrobaculum* clade, though not quite as invariant as seen in other archaeal or bacterial models. Finally, we find an ultra-conserved *Pyrobaculum*-specific promoter-like sequence across every CRISPR array examined. Taken together, we infer that the CRISPR system is endemic in the *Pyrobaculum* clade, and is unlikely to have been horizontally acquired through independent events for each of its members.

Cas6 is presumed to be responsible for cleavage of pre-crRNA, and through its association with the Cas complex is likely responsible for the association of Cas protein Types with CRISPR array families. Cas6 is believed to be responsible for recognition and cleavage of pre-crRNA (Hale et al., 2008). In Type I complexes, CAScade (Brouns et al., 2008) and aCAScade (Lintner et al., 2011), Cas6 is a co-purifying member of the complex. In Type III systems where Cas6 does not appear to be part of the Cas complex, specific proteins that are members of the complex are required for maturation of crRNA (Hatoum-Aslan et al., 2011). Furthermore, the binding of Cas6 in *Pseudomonas aeruginosa* has been shown to be quite specific (Sternberg et al., 2012), and in *S. solfataricus*, there are five distinct Cas6 proteins possibly specialized for specific repeats (Zhang et al., 2012). Taken together, we suggest that Cas6 mediates the association between Cas protein families and CRISPR array families in *Pyrobaculum* species. This mediation may be by direct participation in the Cas complex (Type I systems), or through an indirect association as suggested for Type III systems.

Our transcriptional data clearly show that the *P. islandicum* Type III-A system is capable of generating mature crRNA from each of its five arrays. This Type III-A system is operating without *cas1, cas2, or csm6*. In *Pyrococcus abyssi*, the Type I-A system generates crRNA (Phok et al., 2011) and is also missing *cas1* and *cas2*. Possibly one or both of these systems has an alternative enzymatic method for incorporating novel spacers without CAS1, or one or both of these systems may be incapable of CRISPR adaptation. The missing *csm6* in *P. islandicum* is equally surprising given that it has been considered essential in the Type III-A (csm) system, the only system present in this species. Establishing if *P. islandicum* is still capable of CRISPR adaptation could be a first step in identifying an alternative mechanism for spacer incorporation.

The classification system authored by Makarova (Makarova et al., 2011) has been instrumental in coordinating diverse efforts across the field of CRISPR research. As we examine new phylogenetic clades in detail, we have both a convenient mechanism for classifying our findings as well as adding variations brought into focus by new groups. In light of our new analyses, the consolidation of *csa1* (described herein as *cas4′*) with *cas4* may not be justified, as this would suggest many *Pyrobaculum* submodule examples with two copies of *cas4* (*cas4′-cas1-cas2-cas4)*. Alternatively, we suggest that the functions of *cas4* and *cas4′* (*csa1*) are distinct in *Pyrobaculum* and should be uniquely classified. Furthermore, we find *csm6* (previously named APE2256) deeply associated with a CRISPR-associated *nurA*-*herA* pair, and not apparently part of the Type III-A module where it is currently classified. Finally, we observe that the *csx1* classification (part of Type III-U) given to the numerous *Pyrobaculum* genes encoding a DXTHG domain (or MJ1666-like protein) may not be optimal; in *Pyrobaculum*, these genes appear to be found among Type I and III systems. Clearly, the unique comparative perspective afforded by *Pyrobaculum* provides numerous opportunities for future discovery.

## Author contributions

David L. Bernick designed and performed the experimental and computational analyses, and wrote the manuscript. Courtney L. Cox provided the analysis of the CRISPR promoter sequence conservation. Patrick P. Dennis provided assistance with the manuscript and collaborative review. Todd M. Lowe provided scientific advising, suggested analyses, and edited the manuscript.

### Conflict of interest statement

The authors declare that the research was conducted in the absence of any commercial or financial relationships that could be construed as a potential conflict of interest.

## Appendix

**Table A1:** **Gene reannotations in *Pyrobaculum species***.

**Locus**	**Function**	**Strand**		
***Pyrobaculum aerophilum***				
PAE0067	*cas3*″	−		
PAE0068	*cas3*	−		
Crispr1		−	39812	40776
PAE0075	*cas6*	−		
PAE0077	*csx1*	+		
PAE0079	*cas4*	−		
PAE0080	*cas2*	−		
PAE0081	*cas1*	−		
PAE0082	*cas4*′	−		
Crispr2		+	45503	46687
PAE0109	*cas10*	−		
PAE0111	*csm5*	−		
PAE0112	*csm4*	−		
PAE0114	*csm3*	−		
PAE0115	*csm2*	−		
PAE0117	*csx1*	−		
PAE0119	*csx1*	+		
PAE0122	*nura*	+		
PAE0124	*csm6*	+		
PAE0126	*csm6*	+		
PAE0128	*hera*	+		
PAE0131	*cas6*	+		
PAE0181	*cas6*	−		
Crispr3		+	95531	101005
PAE0198	*cas4*	−		
PAE0199	*cas2*	−		
PAE0200	*cas1*	−		
PAE0201	*cas4*′	−		
PAE0202	*csx1*	−		
PAE0205	*cas8a2*	−		
PAE0207	*cas3*″	−		
PAE0208	*cas3*	−		
PAE0209	*cas5*	−		
PAE0210	*cas7*	−		
PAE0212	*cas6*	+		
Crispr4	+		268866	269081
Crispr5	−		591745	592220
Crispr6/7	−		1898722	1899654
***Pyrobaculum arsenaticum***				
Pars_1108	*herA*	−		
Pars_1109/10	*csm6*	−		
Pars_1111	*nurA*	−		
Crispr2		+	999187	1001495
Pars_1114	*cmr6*	−		
Pars_1115	*cmr1*	−		
Pars_1116	*cmr5*	−		
Pars_1117	*cmr4*	−		
Pars_1118	*cas10*	+		
Pars_1119	*cmr3*	+		
Pars_1120	*csx1*	+		
Crispr3		+	1012951	1018930
Pars_1121	*cas4*	−		
Pars_1122	*cas2*	−		
Pars_1123	*cas1*	−		
Pars_1124	*cas4*′	+		
Pars_1127	*cas7*	+		
Pars_1128	*cas5*	+		
Pars_1130	*cas3*	+		
Pars_1131	*cas3*″	+		
Pars_1133	*cas6*	+		
Pars_1134	*csx1*	−		
Pars_1145	*cas2*	−		
Pars_1147	*cas4*′	−		
Crispr5		+	1039190	1039289
Crispr6		−	1307876	1308104
***Pyrobaculum calidifontis***				
Pcal_0261	*cas6*	−		
Pcal_0263	*cas1*	−		
Pcal_0265	*cas4*′	−		
Crispr1		−	260542	260703
Pcal_0266	*csx1*	−		
Crispr2		+	264904	265204
Pcal_0270	*csx1*	−		
Pcal_0271	*cmr3*	−		
Pcal_0272	*cas10*	−		
Pcal_0273	*cmr4*	+		
Pcal_0274	*cmr5*	+		
Pcal_0275	*cmr1*	+		
Pcal_0276	*cmr6*	+		
Pcal_0277	*csx1*	+		
Crispr3		−	277746	277908
Pcal_1267	*cas3*″	−		
Pcal_1268	*cas3*	−		
Pcal_1270	*cas5*	−		
Pcal_1271	*cas7*	−		
Pcal_1273	*csx1*	−		
Pcal_1274	*cas4*′	+		
Pcal_1275	*cas1*	+		
Pcal_1276	*cas2*	+		
Pcal_1277	*cas4*	+		
Crispr4		−	1185256	1185816
Pcal_1278	*cas6*	−		
Crispr5		−	1188156	1190531
Pcal_1280	*csx1*	−		
Pcal_1281	*csm6*	−		
Pcal_1283	*cmr3*	−		
Pcal_1284	*cas10*	−		
Pcal_1285	*cmr4*	+		
Pcal_1286	*cmr1*	+		
Pcal_1287	*cmr6*	+		
Crispr6		+	1203351	1205855
Pcal_1294	*nurA*	+		
Pcal_1295	*csm6*	+		
Pcal_1296	*herA*	−		
Crispr7		−	1669194	1669346
***Pyrobaculum islandicum***				
Crispr1		−	34	1216
Crispr2		+	38866	39842
Crispr3		+	1404032	1404192
Pisl_1541	*cas10*	−		
Pisl_1542	*csm5*	−		
Pisl_1543	*csm4*	−		
Pisl_1544	*csm3*	−		
Pisl_1545	*csm2*	−		
Crispr4		−	1413797	1414026
Pisl_1932	*cas6*	−		
Crispr5		−	1756971	1759456
***Pyrobaculum oguniense***				
Crispr1			937975	938897
Pogu_1100	*cas4*′	+		
Pogu_1101	*cas1*	+		
Pogu_1102	*cas2*	+		
Pogu_1106	*cas6*	+		
Crispr2			945613	946605
Crispr3			952361	953217
Pogu_1118	*cmr6*	−		
Pogu_1119	*cmr1*	−		
Pogu_1125	*cas10*	−		
Pogu_1126	*csm5*	−		
Pogu_1127	*csx1*	−		
Pogu_1128	*csm3*	−		
Pogu_1135	*csx1*	+		
Pogu_1138	*cas6*	−		
Pogu_1143	*cas8a2*	−		
Pogu_1144	*cas3*″	−		
Pogu_1145	*cas3*	−		
Pogu_1146	*cas5*	−		
Pogu_1147	*cas7*	−		
Pogu_1149	*csx1*	−		
Pogu_1150	*cas4*′	+		
Pogu_1151	*cas1*	+		
Pogu_1152	*cas2*	+		
Pogu_1153	*cas4*	+		
Crispr4			986121	987397
Pogu_1154	*csx1*	−		
Pogu_1155	*cmr3*	−		
Pogu_1156	*cas10*	−		
Pogu_1157	*cmr4*	+		
Pogu_1158	*cmr5*	+		
Pogu_1159	*cmr1*	+		
Pogu_1160	*cmr6*	+		
Crispr5			999562	1002179
Pogu_1165	*nurA*	+		
Pogu_1166/7	*csm6*	+		
Pogu_1168	*herA*	+		
***Pyrobaculum neutrophilum***				
Crispr1		+	511830	513709
Tneu_0562	*cmr3*	−		
Tneu_0563	*cas10*	−		
Tneu_0564	*cmr4*	+		
Tneu_0565	*cmr5*	+		
Tneu_0566	*cmr1*	+		
Tneu_0567	*cmr6*	+		
Tneu_0572	*csx1*	+		
Crispr2		−	526375	526738
Tneu_0576	*cas4*	−		
Tneu_0577	*cas2*	−		
Tneu_0578	*cas1*	−		
Tneu_0579	*cas4*′	−		
Crispr3		+	530828	531454
Crispr4		+	849844	851759
Crispr5		+	856227	857471
Crispr6		+	883097	885730
Tneu_0994	*cas7*	+		
Tneu_0995	*cas5*	+		
Tneu_0997	*cas3*	+		
Tneu_0998	*cas3*″	+		
Tneu_0999	*cas6*	+		
Crispr7		+	994025	995068
Tneu_1114	*cas4*	+		
Tneu_1128	*cas6*	+		
Crispr8		+	1017598	1019142
Tneu_1132	*cas8a2*	−		
Tneu_1133	*cas3*″	−		
Tneu_1134	*cas3*	−		
Tneu_1135	*cas5*	−		
Tneu_1136	*cas7*	−		
Tneu_1138	*csx1*	−		
Tneu_1139	*cas4*′	+		
Tneu_1140	*cas1*	+		
Tneu_1141	*cas2*	+		
Tneu_1142	*cas4*	+		
Crispr9		−	1030559	1032170
Tneu_1143	*nurA*	+		
Tneu_1144	*csm6*	+		
Tneu_1145	*herA*	−		
Crispr10		+	1035988	1038486
Tneu_1149	*csm3*	+		
Tneu_1150	*csm4*	+		
Tney_1151	*csm5*	+		
Tneu_1152	*cas10*	+		
Tneu_1154	*csx1*	+		

**Table A2:** **NurA and HerA paralogs in *Pyrobaculum* species**.

**NurAFamily**	**Pfam Evalue**	**HerA Family**	**Blastp Evalue**
PAE0122	2.2E-16	PAE0128	7.0E-06
Pcal_1294	7.9E-11	Pcal_1296	4.0E-05
Pisl	NA	Pisl_	NA
Pars_1111	5.3E-12	Pars_1108	4.0E-05
Pogu_1165	7.3E-11	Pogu_1168	1.0E-05
Tneu_1143	1.4E-09	Tneu_1145	2.0E-05
Vdis_1157	5.0E-07	Vdis_1158	3.0E-05
			
PAE2154	3.3E-43	PAE2155	9.0E-05
Pcal_1069	1.1E-28	Pcal_1070	1.0E-05
Pisl_0942	2.9E-33	Pisl_0941	8.0E-06
Pars_0817	2.5E-31	Pars_0816	3.0E-05
Pogu_1515	2.4E-31	Pogu_1516	6.0E-05
Tneu_1343	1.5E-31	Tneu_1344	4.0E-05
Vdis_1272	1.2E-18	Vdis_1306	5.0E-12
			
PAE2902	1.6E-65	PAE2903	5.0E-40
Pcal_0359	6.1E-47	Pcal_0358	3.0E-38
Pisl_0299	1.7E-43	Pisl_0300	1.0E-37
Pars_0982	1.2E-45	Pars_0983	4.0E-40
Pogu_1338	2.9E-46	Pogu_1337	5.0E-40
Tneu_1822	1.9E-50	Tneu_1821	2.0E-38
Vdis_0977	5.0E-21	Vdis_0630	1.0E-34
			
		PAE2998	9.0E-23
		Pcal_1112	4.0E-22
		Pisl_0395	1.0E-24
		Pars_1361	4.0E-22
		Pogu_0853	7.0E-22
		Tneu_1732	5.0E-21
		Vdis_0944	3.0E-19

E-values for NurA family paralogs are established using Pfam 26.0 (November 2011) (Punta et al., 2012); E-values for HerA family paralogs are established with Blastp (Altschul et al., 1990), using Sulfalobus solfataricus HerA (SSO2251) as the query and the specific species as the target (wordsize 2, Blosum45 score matrix, Gap existence 13, Gap extension 3). The CRISPR-associated NurA-HerA paralogs are shown in red, and the putative ortholog of NurA-HerA involved in homologous recombination is shown in blue.
